# No association between perinatal mood disorders and hypertensive pregnancies

**DOI:** 10.3389/fpsyt.2022.898003

**Published:** 2022-08-12

**Authors:** Sarah Araji, Ashley Griffin, Wondwosen Kassahun-Yimer, Laura Dixon, Shauna-Kay Spencer, Sheila Belk, Gail Ohaegbulam, Kedra Wallace

**Affiliations:** ^1^Department of Obstetrics and Gynecology, University of Mississippi Medical Center, Jackson, MS, United States; ^2^Program in Neuroscience, School of Graduate Studies in Health Sciences, University of Mississippi Medical Center, Jackson, MS, United States; ^3^John D. Bower School of Population Health, Department of Data Science, University of Mississippi Medical Center, Jackson, MS, United States; ^4^Department of Psychology, University of Mississippi, Oxford, MS, United States; ^5^Department of Pharmacology and Toxicology, University of Mississippi Medical Center, Jackson, MS, United States; ^6^Myrlie Evers-Williams Institute for the Elimination of Health Disparities, University of Mississippi Medical Center, Jackson, MS, United States

**Keywords:** anxiety, depression, interleukin-17, social determinants of health, stress, tumor necrosis factor

## Abstract

Mental health disorders such as anxiety and/or depression are the most common mental health disorders seen among reproductive aged women and can increase during pregnancy. Many sociodemographic risk factors have been associated with anxiety and/or depression in pregnancy, which can lead to adverse maternal and infant outcomes including the risk of a hypertensive pregnancy. The current study prospectively examined self-reported anxiety, depression and stress in pregnant women without a history of fetal loss or mood disorders beginning at 20–26 weeks. At each study visit, circulating immune factors associated with perinatal mood disorders were measured in blood samples that were collected. A total of 65 women were eligible for data analysis, 26 of which had hypertensive pregnancies. There was not a significant difference in self-reported depression, anxiety or stress between hypertensive disorders of pregnancy and normotensive women. Black women were more likely to have a hypertensive pregnancy and develop a perinatal mood disorder compared to non-black women. Both the inflammatory cytokines interleukin-17 and tumor necrosis factor-alpha were increased in patients with perinatal mood disorders. However, additional research is needed in a larger sample to truly understand the relationship between these factors along with the underlying etiologies and the associated outcomes.

## Introduction

Maternal mental health is a significant public health challenge, with 10% of pregnant women and 13% of postpartum women experiencing a mental health problem. Pregnancy causes several transformations in women’s bodies including an increased hormonal environment that contributes to a myriad of psychological and physical health changes. Maternal mental health has recently gained more research interest given the association with adverse maternal and fetal outcomes. For instance, preterm labor, low birth weight and fetal growth restriction have all been associated with anxiety or depression during pregnancy (1, 2). Perinatal mood and anxiety disorders (PMADs) are the most common mental health disorders seen in women of reproductive age (3). They have an impact on maternal and fetal wellbeing as well as adverse outcomes in children (4).

The pathogenesis of PMADs are complex and encompass a wide group of risk factors including history of mental disorders, social determinants of health and biological risk factors (2). Multiple studies have consistently found that depression in the antenatal period is a strong risk factor for developing postpartum depression (5) and that hypertensive disorders of pregnancy (HDP) can lead to physiological changes that might play a role in triggering anxiety and/or depressive episodes (2). Previous research has also found that women who have anxiety disorders or depression, diagnosed either before or during pregnancy are at an increased risk of developing a HDP (6, 7). However, these studies have always examined populations of women with a low incidence of HDP, so the directionality of HDP or PMAD is hard to determine. Therefore, the objective of the current study was to determine the relationship between PMAD and HDP in a population of women who are at an increased environmental risk for developing a HDP. According to the Center for Diseases Control Mississippi, a primarily rural state located in the Southern portion of the United States, is constantly ranked #1 or #2 in the United States for hypertension related deaths and the number of hypertensive adults (43.6% of adult population is hypertensive). The high rate of hypertension in Mississippi, makes this an ideal environment to evaluate the relationship between HDP and PMADs. Based on prior research, we investigated whether women with evidence of PMADs are at an increased risk of experiencing a HDP or worse labor outcomes. We hypothesize that women who have increased antepartum PMADs will have a HDP and more immune activation compared to women without antepartum anxiety and/or depression.

## Materials and methods

### Study design

This was a prospective study approved by the Institutional Review Board (IRB) at the University of Mississippi Medical Center in Jackson, MS. Participants were recruited from August 2018 to June 2020 from outpatient clinics affiliated with the University. Written informed consent was obtained from all study participants who met the inclusion/exclusion criteria. Women between 18 and 40 years of age were eligible if they were between 20 and 26 weeks pregnant at recruitment with a single fetus free from fetal anomalies. Participants were excluded if they had a history of stillbirth, fetal death or loss of a child, history of alcohol or drug abuse, HIV/AIDS, history of preterm birth, or history of a high-risk pregnancy (i.e., preeclampsia; PreE). Also to ensure that we were only examining women with new cases of anxiety/depression a history or current diagnosis of anxiety/depression and/or a psychosis was also an exclusion factor. All of these factors have been reported to contribute to the development of PMAD so they were excluded for this initial study. In the current analysis women who met or exceeded the cut-off on either the PASS or EPDS were categorized as PMAD.

### Procedure and instruments used to evaluate stress, depression and anxiety

**FIGURE 1:**
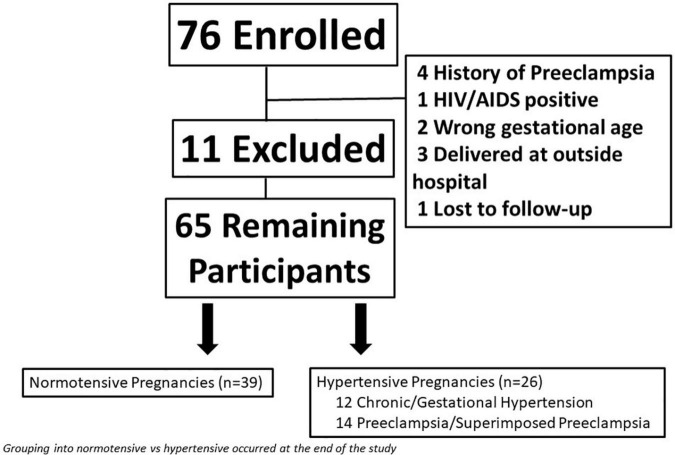
Flow diagram of total study participants including women who were excluded from the study.

As outlined in the Figure 1, women completed two study visits (recruitment and 10 weeks post-recruitment) and a series of validated questionnaires were administered to assess stress (Perceived Stress Scale; PSS), depression (Edinburgh Postnatal Depression Scale; EPDS) and anxiety (Perinatal Anxiety Screening Scale; PASS) were completed.

• The PSS is a validated 14-item self-reported questionnaire used to assess one’s perception of the extent of stress in one’s life. Respondents rate the frequency of their feelings and thoughts about life situations over the month using a five-point Likert-type scale (0-never, 4–very often). This survey is not used for diagnosis but as a clinical screening tool for stress. Items are summed, and a score between 14 and 26 indicates moderate stress and 27–40 indicates high levels of stress (8).
• The EPDS is a clinical screening tool validated to screen for depression both during pregnancy and in the postpartum period (9). It is a 10-item self-report questionnaire in which women rate their feelings over the past 7 days. Each question is scored 0–3 (score range 0–30) and it is quick to complete. The cut-off value of >12 was used for probable depression (10).
• The PASS is a validated 38-item self-report questionnaire used to screen for anxiety over the past month among pregnant women. Respondents rate symptoms of acute anxiety and adjustment, general worry and specific fears, perfectionism, trauma and control, and social anxiety on a four-point Likert-type scale (0-not at all, 3-all the time). The cut-off scores for mild to moderate anxiety is 0–21, moderate to severe 21–41 and 42–93 for severe anxiety (11, 12).

Women who exceeded the cut-off scores were provided feedback on their study results and offered additional mental health resources outside of the study.

Participants also completed a survey of sociodemographic characteristics (i.e., employment and relationship status, education level). To evaluate socioeconomic status, we utilized the distressed community index (DCI). The DCI measures economic wellbeing at a zip code level by assessing location based disparities and evaluating the relationship between them and health outcomes, demographics and educational level (13). Communities are scored and divided into five tiers based on these distressed levels: prosperous (<20.0), comfortable (20.1–40.0), mid-tier (40.1–60.0), at risk (60.1–80.0) and distressed (> 80.0).

### Data entry and collection

Health data was extracted from electronic medical records, and all data were stored in REDCap (14). The following electronic health data was collected: residential zip code, medical history including details on obstetric characteristics; body mass index (BMI), labor and delivery details, and final hypertensive status (e.g., PreE, gestational hypertension). Intrapartum complications such as fever, postpartum hemorrhage, and shoulder dystocia were also collected. From infant records the following was collected: birth weight and information pertaining to presence of infant complications at birth.

### Study groups

After all women had completed the study, participants were grouped based on the hypertensive status at the time of discharge from labor and delivery. Women were categorized as normotensive pregnancy (*n* = 39) or hypertensive pregnancy (*n* = 26). Women were considered to have a hypertensive pregnancy if they had chronic hypertension (cHTN), gestational hypertension (gHTN), PreE or super-imposed PreE (siPreE).

### Blood collection and quantification of circulating cytokines

At each study visit blood was collected by a study nurse or a member of the facility laboratory. Samples were collected in EDTA (BD, United States) and serum collection tubes and allowed to clot. Tubes were centrifuged at 2,000 RPM at 4°C for 20 min, aliquoted and frozen at −20°C. Maternal plasma levels of specific cytokines were measured using commercially available Human Premixed Multi-Analyte Magnetic Luminex Assay (R&D systems, United States) and assayed in duplicate according to the manufacturers recommendations. Multiplex panels were designed for the following proteins and cytokines, all of which are associated with perinatal anxiety and depression or hypertensive pregnancy (15–17): soluble fms-like tyrosine kinase-1 (sFlt-1), soluble endoglin (sEng) and placental growth factor (PlGF). All Luminex assays were read and analyzed via the Luminex MAGPIX analyzer. Interleukin-17 (IL-17, Boster Bio, CA, United States) and tumor necrosis factor-alpha (TNF-α; RayBiotech, GA, United States) were measured via individual ELISAs according to the manufacturer’s protocol.

### Data analysis

Summary statistics were presented as means (standard deviations) for continuous variables and using frequencies and percentages for categorical variables. Correlation among continuous variables was estimated using Pearson’s correlation coefficient. Chi-square test, Fisher’s exact tests, *T*-test were used to compare participant characteristics between HDP and Normotensive categories. Similar statistical tests were used to compare demographic characteristics of women among PMAD and non-PMAD categories. Multivariable adjusted logistic regression models were used to assess associations between HDP, maternal age, maternal BMI and race. Data for EPDS, PASS and PSS scores were presented at Visit 1 and Visit 2 for all women as well as by HDP and Normotensive categories. All statistical analyses were performed with SAS version 9.4 (SAS Institute Inc., Cary, NC, United States). A two-sided *p* < 0.05 was considered statistically significant.

## Results

A total of 76 women enrolled in the study. Eleven women were removed for either delivering at an outside hospital (*n* = 3), lost to follow-up (*n* = 1), or did not meet inclusion/exclusion criteria upon chart review (*n* = 7); leaving a total of 65 women remaining in the study (Figure 1). Eight women were not seen at visit 2 because they had a preterm delivery.

### Women who were black, older and obese were more likely to experience a hypertensive disorders of pregnancy

The baseline sociodemographic characteristics of study participants participating in the study are shown in Table 1. Women who had a HDP were significantly older (*p* = 0.006) and had a higher BMI (41.9 ± 13.5 kg/m^2^, *p* = 0.0002) relative to women with normotensive pregnancies. There was a significant difference in the racial distribution between women experiencing HDP and normotensive pregnancies (*p* = 0.0007). As shown in Table 1, black women comprised 84.5% of HDP while white women comprised 51.2% of normotensive pregnancies. Multiple logistic regression showed that women who were older (*p* = 0.02), had a higher BMI (*p* = 0.01) or who were of black race (*p* = 0.01) had significant odds of having a HDP (Supplementary Table 1).

**TABLE 1:** Sociodemographic characteristics of study participants at baseline (visit 1).

	Normotensive (*n* = 39)	HDP (*n* = 26)	*P-value*
Age (years)	25.97 ± 4.36	29.57 ± 5.87	0.006
Maternal race (%) Black White Other	46.2 51.2 2.6	84.5 11.5 3.8	0.0007
Gestational age (weeks)	23.07 ± 2.02	23.72 ± 1.7	0.19
BMI (kg/m^2^)	31.34 ± 8.09	41.9 ± 13.5	0.0002
Education (%) High school Diploma/GED Some college Technical/community college degree Bachelor’s degree Master’s degree or beyond	31.6 26.3 13.2 10.5 18.4	18.5 37.0 18.5 14.8 11.2	0.61
Relationship (%) Married Single In a relationship Other	56.4 12.8 30.8 0	30.8 34.6 23.1 11.5	0.01
Employment Employed FT/PT/on leave/student Not currently working Homemaker/disabled	71.1 15.7 13.2	59.3 33.3 7.4	0.17
Distressed community index (%) Prosperous Comfortable Mid-tier At-risk Distressed	7.7 15.4 7.7 15.4 53.8	3.8 7.7 3.8 19.2 65.5	0.76

There was not a significant difference in self-report There was a significant difference in relationship status, as women who had a HDP were less likely to be married and more likely to be single compared to women with a normotensive pregnancy (*p* = 0.01). However, no differences were found between the groups in relation to education level (*p* = 0.61), current employment status (*p* = 0.17) or their DCI (*p* = 0.76).

### Women with a hypertensive disorders of pregnancy delivered earlier and had lower birth weight infants

Women with a HDP gave birth significantly earlier (35.59 ± 3.17 weeks) than normotensive women (38.04 ± 2.15 weeks, *p* = 0.0004; Table 2). This corresponded to a higher incidence of preterm birth (*p* = 0.0001) and a significantly lower infant birth weight among women with HDP (*p* = 0.009; Table 2). Despite the higher rate of preterm delivery there were no differences in mode of delivery (*p* = 0.12), maternal labor complications (*p* = 0.34) or infant complications (*p* = 0.25) between the two groups of women.

**TABLE 2:** Birth outcomes between women with normotensive pregnancies and hypertensive disorders of pregnancy (HDP).

	Normotensive (*n* = 39)	HDP (*n* = 26)	*P-value*
Gestational age at delivery (weeks)	38.04 ± 2.15	35.59 ± 3.17	0.0004
Preterm birth (%) No Yes	81.6 18.4	33.3 66.7	0.0001
Mode of delivery (%) Vaginal Cesarean section	67.57 34.2	46.15 53.85	0.12
Labor complications (%) No Yes	69.23 30.77	80 20	0.34
Birthweight (g)	3226.7 ± 591.3	2781.3 ± 721	0.009
Infant complications (%) No Yes	91.4 8.6	76.4 23.8	0.25

### Women with hypertensive disorders of pregnancy did not have increased depression, anxiety or perceived stress

There was not a significant difference in self-reported depression, anxiety or stress between HDP and normotensive women at either visit 1 or visit 2. Women were also ranked as low depression vs. probable depression based on their EPDS value. Although 16.9% of women were at risk for probable depression, no significant differences were observed between normotensive and HDP pregnancies when this relationship was further examined (*p* = 0.99, Table 3). Results from the PASS was used to evaluate minimal anxiety vs. moderate/severe anxiety among women with normotensive and HDP pregnancies at visits 1 and 2. At both visits women with normotensive pregnancies were more likely to screen with moderate/severe anxiety relative to women with a HDP (*p* = 0.11 at visit 1; *p* = 0.07 at visit 2).

**TABLE 3:** Scores for EPDS, PASS, and PSS were divided into different categories based on cut-off scores and the number of women were compared between normotensive pregnancies and hypertensive disorders of pregnancy (HDP).

	All women	Normotensive (*n* = 39)	HDP (*n* = 26)	*P-value*
**EPDS (%)** Visit 1 Low risk (0–11) Probable depression (12–30) **Visit 2** Low risk (0–11) Probable depression (12–30)	83.1 16.9 84.2 15.8	84.21 15.79 85.29 14.71	81.48 18.52 82.61 17.39	0.99 0.99
**PASS (%)** Visit 1 Minimal (0–20) Moderate/Severe (21–93) **Visit 2** Minimal (0–20) Moderate/Severe (21–93)	65.6 34.4 73.7 26.3	56.76 43.24 64.71 35.29	77.78 22.22 86.96 13.04	0.11 0.07
**PSS (%)** Visit 1 Minimal (0–13) Moderate/Severe (14–40) **Visit 2** Minimal (0–13) Moderate/Severe (14–40)	9.2 90.8 19.3 80.7	5.26 94.74 11.76 88.24	14.81 85.19 30.43 69.57	0.22 0.09

The point range for each categorization within the instruments is listed in parentheses.

Likewise, perceived stress decreased in both group of women between visits 1 (*p* = 0.22) and 2 (*p* = 0.09), with normotensive women being more likely to report higher levels of stress relative to women with a HDP (Table 3).

Among HDP women, 53.8% (*n* = 14) women had PreE vs. a diagnosis of cHTN/gHTN (*n* = 12). There were no statistically significant differences between women in any of the demographic factors with the exception of BMI. Women who developed PreE/siPreE were significantly heavier at the time of enrollment (48.14 ± 15.1 vs. 36.12 ± 8.5 kg/m^2^; Supplementary Table 2) compared to women with cHTN/gHTN. When labor complications were examined, women with PreE/siPreE were more likely to have a preterm delivery relative to women with cHTN/gHTN (78.6 vs. 25%, *p* = 0.02; Supplementary Table 2). However, there were not any significant differences between any other variables. Women with PreE/siPreE were also not more likely to have a PMAD at either visit 1 (*p* = 0.99) or visit 2 (*p* = 0.99).

### Perinatal mood and anxiety disorder was not associated with lower socioeconomic factors or worst birth outcomes

In the current analysis there 27 women were categorized as PMAD and compared to 38 non-PMAD women. There was not a significant relationship between HDP and PMAD at either visit 1 (*p* = 0.21) or visit 2 (*p* = 0.07). The overall percent of women with a PMAD decreased 16% (*p* = 0.06) between visit 1 and visit 2 regardless of their hypertensive status, with no significant changes in outcomes due to a change in PMAD status.

No significant differences were found in baseline demographic factors between women who had a PMAD and those who did not (Table 4). However, when race was collapsed into black vs. non-black, black women were 40% more likely to report a PMAD, O.R. 1.39 95% CI (0.54–3.63) relative to non-black women. Current employment status (*p* = 0.39) or education level (*p* = 0.62) did not have an impact on PMAD, with the majority of women experiencing a PMAD (59%) having completed a high school diploma/GED or some college (Table 4). Relationship status also had no significant impact on PMAD (*p* = 0.11). DCI was not associated with PMAD (*p* = 0.12) as the majority of women lived in at risk or distressed communities regardless of their PMAD status. Experiencing a PMAD was not associated with an increased gestational age at delivery (*p* = 0.51), pre-term birth (*p* = 0.43) or decreased infant birth weight (*p* = 0.53, Table 4). Similarly women with PMAD did not experience more labor complications (*p* = 0.15) or infant complications (*p* = 0.71) relative to women without PMAD (Table 4).

**TABLE 4:** Sociodemographic and labor characteristics between women with and without perinatal mood and anxiety disorders (PMAD).

	Non-PMAD (*n* = 38)	PMAD (*n* = 27)	*P-value*
Age (years)	28.34 ± 5.2	26.37 ± 5.38	0.14
Maternal race (%) Black White Other	55.3 39.5 5.2	70.4 29.6 0	0.30
BMI (kg/m^2^)	35.65 ± 11.97	35.44 ± 11.5721	0.94
Education (%) High school diploma/GED Some college Technical/community college degree Bachelor’s degree Master’s degree or beyond	23.6 31.6 13.1 10.5 21.2	29.6 29.6 18.5 14.8 7.5	0.62
Relationship (%) Married Single In a relationship Other	57.8 15.8 21.1 5.3	29.6 29.6 37.0 3.8	0.11
Employment Employed FT/PT/on leave/student Not currently working Homemaker/disabled	65.8 21.0 13.2	66.7 25.9 7.4	0.39
Distressed community index (%) Prosperous (below 20) Comfortable (20.0–40.0) Mid-tier (40.1–60.0) At Risk (60.1–80.0) Distressed (80.1–100)	5.2 7.9 10.5 23.7 52.7	7.4 18.5 0 7.4 66.7	0.12
Gestational age at delivery (weeks)	36.86 ± 2.8	37.34 ± 2.95	0.51
Preterm birth (%) No Yes	63.1 36.9	74 26	0.43
Mode of delivery (%) Vaginal Cesarean section	63.2 36.8	44.4 55.6	0.21
Labor complications (%) No Yes	81.8 18.2	63 37	0.15
Birthweight (g)	2992.4 ± 747.1	3102.3 ± 585.6	0.53
Infant complications (%) No Yes	89.2 10.8	85.2 14.8	0.71

### Perceived stress was higher in women with perinatal mood and anxiety disorders

**FIGURE 2:**
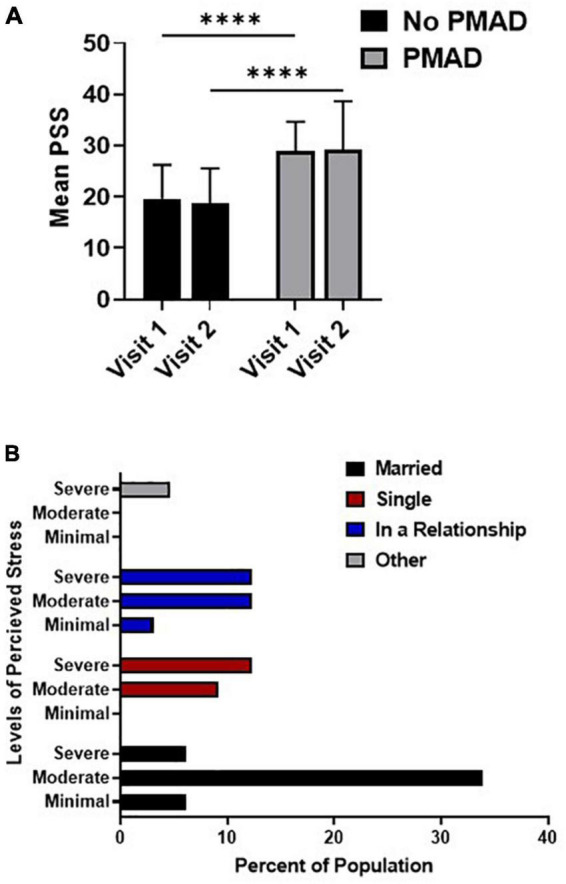
Relationship between perceived stress and PMAD showed significance at both visits **(A)** while the association of relationship status and PSS was analyzed in **(B)**. ^∗∗∗∗^Denotes *p* < 0.00005. PMAD, Perinatal mood and anxiety disorders; PSS, Perceived stress scale.

A significant relationship was found between PSS and PMAD, as women with PMADs had significantly higher levels of PSS at both visit 1 (*p* < 0.0001) and visit 2 (*p* < 0.0001) relative to women without PMADs. Additionally, the mean level of PSS did not significantly change between visit 1 and visit 2 among women with (*p* = 0.99) or without PMADs (*p* = 0.94; Figure 2A). To further explore the impact of perceived stress, we examined the levels of perceived stress and demographic factors.

At both visit 1 (*p* = 0.006) and visit 2 (*p* = 0.03) women who were married were more likely to experience moderate levels of stress, while non-married women in relationships or who were single were more likely to experience severe levels of stress (Figure 2B). Interestingly, a significant relationship was found between PSS and DCI at visit 2 (*p* = 0.02), however, upon further examination it was found that 68.9% of the women (22/32) living in distressed zip codes reported moderate-severe stress. However, PSS was not significantly associated with any other socioeconomic or demographic factors nor was it associated with labor or birth outcomes (Supplementary Data).

### Women with perinatal mood and anxiety disorders have increased IL-17 and TNFα

**FIGURE 3:**
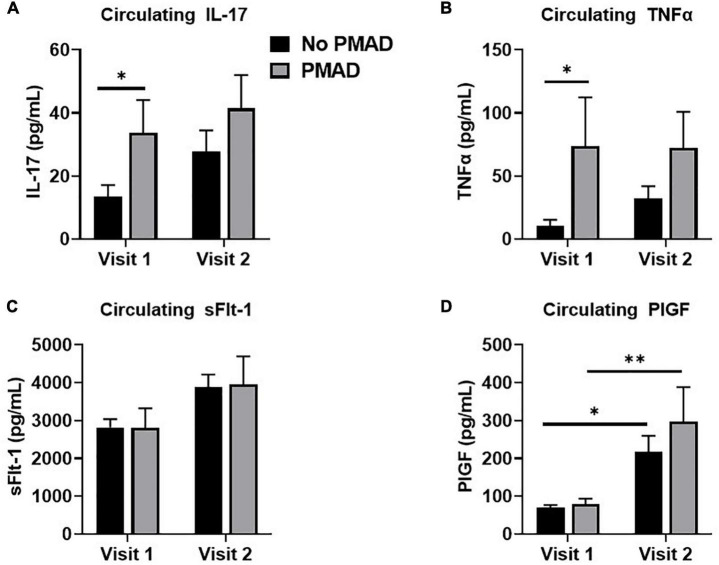
Measurement of circulating levels of IL-17 **(A)**, TNFα **(B)**, sFlt-1 **(C)**, and PLGF **(D)** showed varying levels across visits for women with PMAD. ^∗^ and ^∗∗^denotes *p* < 0.05–0.005.

Biological variables were assessed to determine if they differed in the 2nd or 3rd trimester among women with and without PMADs. At visit 1 there was a significant difference in IL-17 levels between women with and without PMADs (*p* = 0.03, Figure 3A). Circulating levels of TNFα were also found to be significantly increased (*p* = 0.009; Figure 3B), with significant differences at visit 1 (*p* = 0.04). We also examined, anti-angiogenic growth factors that are commonly associated with a HDP to see if they changed and we did not see any significant differences between groups in circulating soluble endoglin (*p* = 0.12). There was not a group effect on circulating sFlt-1 levels, but there was a time effect as sFlt-1 significantly increased between visit 1 and visit 2 regardless of maternal mood (*p* = 0.01, Figure 3C). The same effect occurred with circulating PlGF levels, which increased as pregnancy increased (*p* < 0.0001, Figure 3D).

## Discussion

Maternal mental health is a critical component of a healthy pregnancy. PMADs have a complex pathogenesis with evidence suggesting that physiological changes triggered by HDP may play a causal role in anxiety and/or depressive symptoms (2). Studies examining women with a HDP have found associations between HDP and PMADs and a correlation with adverse birth outcomes (1, 18). In the current study, our objective was to determine if there is a relationship between PMADs and HDP in a population of women living in Mississippi.

Despite the high prevalence of HDPs in our sample, PMAD was not associated with HDP. These findings are in contrast to findings from previous studies (19). For instance, Dachew et al., examined a large cohort of women and found that women who developed PreE had a 53% increased risk of experiencing antenatal depression relative to women who did not have PreE (20). Similar results were observed when HDP and PMADs were examined in a population-based retrospective study (21). However, when models were adjusted for sociodemographic factors, anxiety was the only mental health disorder associated with hypertension during pregnancy. It is important to note that although an association between HDP and PMAD was not found in the current study, several findings are consistent with the literature. For instance, our data indicated that as maternal age and BMI increased the risk of HDP increased, which has been reported by multiple studies (22, 23). Additionally, black women in our sample were much more likely to develop HDP and to experience a PMAD vs. non-black women (24, 25).

There was not a significant increase rate in the number of cesarean deliveries or infant complications in the HDP patients as noted in previous studies (26); however, women with HDP had babies with significantly lower birth weights which is consistent with the literature (27, 28). Adverse pregnancy outcomes are common among women with HDPs. A systematic review and meta-analysis examining outcomes among pregnant women with chronic hypertension reported a RR of 2.7 (1.9–3.6) for preterm delivery < 37 weeks gestation and an RR of 1.3 (1.1–1.5) for cesarean section (26). Another study investigating perinatal outcomes in women with severe hypertension reported a mean gestational age of 34.5 ± 4.6 weeks at delivery (29), again indicative of pre-term birth. The findings of HDP being associated with preterm birth is supportive of what was found in our patient sample. A large number of studies have investigated the association between PMADs and birth outcomes. Prior studies have found that preterm birth increased in patients with high levels of pregnancy related anxiety (RR) = (2.1, 95% CI: 1.5, 3.0), as was depression during pregnancy (aOR) = 1.71; 95% CI (1.65–1.77) (30, 31).

We examined certain social determinants of health as both social determinants of health and perceived social support are key factors contributing to maternal mental health (2, 32). We did not find an association between employment status and HDP, however, married women were less likely to have a HDP, which is consistent with studies showing that partner/social support is protective against HDP (33).

There were no significant correlations between age and PMAD or DCI and PMAD. To further the contrast between our sample and previous research, there was not an association between PMAD and relationship status, education level or employment status. In contrast, previous studies have shown that being in a relationship, employed, and/or achieving higher levels of education can be protective against anxiety and depression during the perinatal period (34–37).

As stress can be related to socioeconomic and sociodemographic factors we sought to evaluate the associations between perceived stress and selected factors. When we looked at PSS scores and relationship status, married women were more likely to have moderate levels of stress, and women who were single or unmarried while in a relationship were more likely to have severe levels of stress. This finding is consistent with previous studies, which have reported that partner support is protective against stress (36, 37). Our population was different in that there was no significant difference in stress levels between women who were employed vs. those who were unemployed. This contradicts a previous study where unemployed women were found to have higher levels of stress (35). The inconsistency between our population and the literature could be attributed to differences in race, geographical locations, and/or culture.

To determine if there was a difference in inflammatory markers among women with and without PMADs, we evaluated the correlation between immune circulating factors and comorbidity and HDP. Both IL-17 and TNFα, were found to be increased in women with PMADs compared to women without PMAD. This result is consistent with previous studies reporting increased levels of IL-17 in patients with depression (38). In addition, TNFα levels were increased in patients with PMADs; whereas no PMAD relationship was found with sFlt-1 and PlGF. These results suggest that there is a direct relationship between IL-17, TNFα, and PMADs during pregnancy. It has been suggested that IL-17 can cause depression by promoting the production of other pro-inflammatory cytokines such as TNFα which ultimately can interfere with serotonin production (39–41).

Our study had several limitations. For instance, research personnel were prohibited from attending some clinics during the height of the COVID-19 pandemic in 2020. This led to missed blood draws for a small portion of study participants (*n* = 6). In addition, all women in the current study were free from a history of infant loss, PreE, or a history of anxiety and/or depression (42, 43). This criterion may have contributed to a decreased risk of PMAD, and the current results may represent an underestimate. However, despite the strict inclusion/exclusion criteria, and the small number of women in the study, the study was adequately powered (> 99%) based on the primary objective to determine a relationship between HDP and PMAD.

To conclude, the current study did not find an association between PMAD and HDP. Patients in distressed zip codes are more stressed, suggesting that socioeconomic factors are tightly linked to mental health. Black women were more likely to have a HDP and develop a PMAD compared to non-black women.

Although the data from our study conflicts with existing literature, one possible explanation for our findings may be within our sample population. Studies have indicated that race, resilience and coping mechanisms all contribute to mental health and cardiovascular outcomes in black individuals (44, 45). It is important to note that the majority of our study sample was composed of non-Hispanic Black women and women who lived in areas considered to be at-risk or distressed. As such, it could be that women in our study are more resilient to variables that are typically considered as stressors. This can lead to a better adaptation to potentially distressing situations and the lack of resources which might have an undesirable effect on mental health and ultimately contribute to adverse pregnancy outcomes. Though there are several factors that can contribute to PMADs or even the self-reporting of PMADs it is important that mental health continue to be evaluated for women during pregnancy and the postpartum period (46). Importantly as studies have indicated that adverse experiences and childhood traumatic events are more frequently recalled during the ante and postpartum period it is important to recognize the utility and administration of self-report exams/surveys among populations that may be at risk (47). Current studies by our group are building on the study design in the present report with the integration of self-reported adverse childhood experience and brief resilience surveys and including women with a history of PreE or depression/anxiety into the study. However, to truly understand the relationship of HDP, sociodemographic factors and PMAD along with the underlying etiologies and the associated outcomes, a larger more diverse samples must be studied.

## Data availability statement

The original contributions presented in this study are included in the article/Supplementary material, further inquiries can be directed to the corresponding author.

## Ethics statement

The studies involving human participants were reviewed and approved by the Institutional Review Board of University of Mississippi Medical Center. The patients/participants provided their written informed consent to participate in this study.

## Author contributions

KW and LD contributed to the conception and design of the research. SA, AG, S-KS, SB, and GO conducted the research which was supervised by KW. WK-Y performed the statistical analysis. SA, KW, and LD interpreted the data. SA wrote the first draft of the manuscript. All authors read and contributed to the editing of this manuscript.
