# Supplementary material for: No association between perinatal mood disorders and hypertensive pregnancies
Source: Front Psychiatry. 2022 Aug 12;13:898003. doi: 10.3389/fpsyt.2022.898003 (PMC9412728; doi:10.3389/fpsyt.2022.898003)
Supplement: Supplementary file 1 [file Data_Sheet_1.docx]

**Supplemental Data**

**Supplemental Table 1**. Multiple logistic regression was performed to determine independent associations between select variables and a hypertensive disorder of pregnancy. The odds ratio estimates and 95% confidence intervals are listed in this table.

| **Effect** | **Point Estimate** | **95% Confidence Limits** | |
| --- | --- | --- | --- |
| **Maternal Age (years)** | 1.17 | 1.03 | 1.32 |
| **BMI (kg/m^2^)** | 1.09 | 1.02 | 1.16 |
| **Black Race** | 5.81 | 1.52 | 22.28 |

**Supplemental Table 2**. Demographic factors for study participants with hypertension were compared.

|  | **cHTN/gHTN**  **(n=12)** | **PreE/SiPreE**  **(n=14)** | **P Value** |
| --- | --- | --- | --- |
| **Age (years)** | 29.25+6.8 | 28.92+5.4 | 0.89 |
| **Maternal race (%)**  Black  Non-Black | 83.3  16.7 | 85.7  14.3 | 0.99 |
| **Nulliparous (%)** | 33.3 | 42.9 | 0.70 |
| **BMI (kg/m^2^)** | 36.12+8.5 | 48.14+15.1 | 0.02 |
| **Education (%)**  High school Diploma/GED  Some college  Technical/Community college degree  Bachelor’s degree  Master’s degree or beyond | 16.6  41.2  16.6  16.6  9 | 35.7  35.7  14.4  7.1  7.1 | 0.83 |
| **Relationship (%)**  Married  Single  In a relationship  Other | 41.6  25  25  8.4 | 21.4  42.9  21.4  14.3 | 0.69 |
| **Employment**  Employed FT/PT/on leave/student  Not currently working  Homemaker/disabled | 50  41.7  8.3 | 64.3  28.6  7.1 | 0.76 |
| **Distressed Community Index (%)**  Prosperous (below 20)  Comfortable (20.0-40.0)  Mid-tier (40.1-60.0)  At Risk (60.1 – 80.0)  Distressed (80.1-100) | 8.3  8.3  0  16.7  66.7 | 0  0  7.1  21.4  71.5 | 0.51 |
| **EPDS (raw value)**  Visit 1  Visit 2 | 6.75+5.2  6.45+5.6 | 5.21+5.76  4.55+5.63 | 0.48  0.43 |
| **PASS (raw value)**  Visit 1  Visit 2 | 17.83+15.18  14.73+14.59 | 13.89+15.04  11.45+12.94 | 0.51  0.58 |
| **PSS (raw value)**  Visit 1  Visit 2 | 24.08+8.6  20.82+8.95 | 22.21+8.87  19.91+10.18 | 0.59  0.83 |
| **PMAD (%)**  No  Yes | 66.7  33.3 | 71.4  28.6 | 0.99 |
| **Gestational age Delivery (weeks)** | 36.65+3.22 | 34.58+3.02 | 0.11 |
| **Preterm delivery (%)** | 25 | 78.6 | 0.02 |
| **Mode of delivery**  Vaginal  Caesarean section | 66.7  33.3 | 33.3  66.7 | 0.22 |
| **Maternal labor complications**  No  Yes | 91.7  8.3 | 64.3  35.7 | 0.17 |
| **Infant complications**  No  Yes | 91.7  8.3 | 71.4  28.6 | 0.33 |

**Supplemental Table 3**. Individual PASS scores at visit 1 were categorized as minimal, mild-moderate and severe. Select sociodemographic factors and labor/birth outcomes were compared between the PASS groups to determine if there was a relationship between severity of anxiety and the variables of interest.

|  | **Minimal**  **(n=6)** | **Moderate**  **(n=36)** | **Severe**  **(n=23)** | **P Value** |
| --- | --- | --- | --- | --- |
| **Maternal age (years)** | 30.67+5.2 | 27.83+5.5 | 26.05+4.92 | 0.15 |
| **Education (%)**  High school Diploma/GED  Some college  Technical/Community college degree  Bachelor’s degree  Master’s degree or beyond | 0  33.3  16.7  16.7  33.3 | 27.8  25  19.4  11.1  16.7 | 30.4  39.1  8.7  13.1  8.7 | 0.59 |
| **Employment (%)**  Employed FT/PT/on leave/student  Not currently working  Homemaker/disabled | 100  0  0 | 63.9  22.2  13.9 | 56.5  34.8  8.7 | 0.29 |
| **Gestational age Delivery (weeks)** | 38.33+1.35 | 36.98+2.75 | 36.89+3.34 | 0.53 |
| **Preterm delivery (%)** | 16.7 | 33.3 | 27.3 | 0.68 |
| **Mode of delivery**  Vaginal  Caesarean section | 50  50 | 57.6  42.4 | 64.2  35.8 | 0.83 |
| **Maternal labor complications (%)**  No  Yes | 83.3  16.7 | 72.2  27.8 | 72.7  27.3 | 0.85 |
| **Birthweight (grams)** | 3206.7+682.6 | 3002+709.3 | 3059+673.1 | 0.79 |
